# Conceptual blending in humans and language models

**DOI:** 10.3389/fpsyg.2026.1849083

**Published:** 2026-07-29

**Authors:** Spencer K. Lynn

**Affiliations:** Division of Human-Centered Artificial Intelligence, Charles River Analytics, Cambridge, MA, United States

**Keywords:** affordances, concept integration, conceptual blending, conceptual flexibility, embodiment, large language models, predictive processing

## Abstract

In theories of embodied cognition, concept integration, or *blending*, integrates features from different source concepts to construct a new, context-sensitive meaning and is considered a core mechanism of human conceptual flexibility. Blending, while a cognitive act, is reflected in language, including that produced by large language models (LLMs). The seeming naturalness of blends produced by language models raises a question about underlying mechanisms of blending in brains versus models, explored in this conceptual analysis. For humans, blending is related to the embodied nature of cognition: we are constrained to perceive the environment in terms of affordances that may achieve our goals. Blending combines perceived affordances into a new concept that can satisfy the goal. I develop a predictive processing account of human generative-causal blends in which goals are parent nodes in a generative hierarchy, predicting affordances that influence perception and categorization. The account is illustrated with a toy example implemented as a vector symbolic architecture in a Python script. In contrast, language models are optimized for next-token prediction and produce distributionally novel blends constrained by proxy-goals that are derived from alignment and prompt framing within the context window. I discuss distinctions bearing on conceptual flexibility, agent autonomy, and their implications for agent design.

## 1 Introduction

### 1.1 The blending puzzle

What do these four things have in common: The creative process of brainstorming novel design requirements (Confalonieri et al., 2018), e.g., for a rock-climber’s hot-beverage container; the enumeration of *ad hoc* or unfamiliar category members (Barsalou, 2016), like “tasks you might skip in the morning so you can get to work on time”; the conventionalized meanings of compound words (Hedblom, 2020), such as “mothership”; and the semantics of mundane prepositional phrases (Ounis, 2021) like “on the table” versus “on time”? In theories of embodied cognition (Fuchs, 2026; Varela et al., 2017), these heterogeneous phenomena can be interpreted through a common lens, concept integration or *blending* (Fauconnier and Turner, 2003). Conceptual integration theory characterizes how the mind blends elements from diverse “mental spaces” (e.g., functionally associated knowledge about concepts, situations, and events) to construct meaning. In the field of cognitive linguistics (Lakoff, 1987), concept blending is considered a fundamental cognitive operation that underpins human intelligence, creativity, and conceptual flexibility because blending integrates elements of existing source concepts to create a new concept or to contextually refine an existing concept, the blend. A distinguishing feature of blending is that a blend is not merely a subtype of a parent concept, nor a union of the source concepts. Rather, a blend is a selective combination of specific elements of each source. For example, the meaning of the conventional blend “house boat” is not the union of necessary and sufficient conditions of houses and boats, as evidenced by contrast with the meaning of another blend, “boat house” (Hedblom, 2020). Instead, the typical meanings of these conventionalized blends are derived from a particular combination of different features from the same two source domains.

Blends may be creative and novel, conventionalized, *ad hoc*, or mundane, but in all cases, a central puzzle about blending is: What motivates the inheritance of specific parent elements into the blend? Related puzzles include, How do brains do it? and What are possible methods of computational implementation? While blending describes capabilities supporting conceptual flexibility of brains reflected in natural language and multimodal human communication, large language models [LLMs and related multimodal variants blend, too (Peng et al., 2025; Sato, 2025)]. Transformer language models contextualize a prompt with their knowledge to generate previously unseen, blended output. Here, I offer a conceptual analysis of answers to these questions, positing that it is one’s goals (sensu, e.g., Barrett and Finlay, 2018) that motivate the blend (Lynn et al., 2024, 2023) via, for brains, an embodied generative-causal mechanism and, for language models, a proxy-goal arising from prompt- and alignment-induced constraints over token generation, consistent with learned statistics relative to the training distribution. If human adaptability is in part due to a capability for blending, then understanding how language models approximate blending and where that differs from human blending provides a way to diagnose when and why artificial intelligence (AI) conceptual flexibility lags human capabilities and may suggest remedies.

### 1.2 Theoretical scope and levels of explanation

This article integrates and contrasts several traditions. Conceptual integration theory is treated primarily as a descriptive and cognitive-linguistic account of selective projection among mental spaces. It characterizes what must be explained when a blend is constructed. Embodied cognition and cognitive linguistics provide an epistemological and representational frame. Concepts are not labels, but patterns of situated sense-making shaped by bodily capacities, image schemas (Lakoff and Johnson, 1980), and force-dynamic relations (Talmy, 1988). Conceptual integration theory does not originate as an embodied cognition theory per se, however I follow an embodied interpretation of it in which selection of source elements is guided by goals, affordances, and possible action, and I offer a predictive interpretation of blending phenomena. Affordance theory (Gibson, 2014) describes the interface between an organism and physical or conceptual environment: what a perceived arrangement of entities affords relative to goals, capacities, and context. Predictive processing (Clark, 2016; Hohwy, 2013) is used here as a candidate mechanistic and computational account of how goal-conditioned predictions and error signals could motivate and stabilize human blends. Language models provide an engineering comparison. They generate blend-like outputs through learned representations, attention, post-training, context conditioning, and decoding. The analysis treats blending as the phenomenon to be explained, embodiment and affordances as an account of why particular projections matter for brains, predictive processing as one proposed implementation for human goal-directed blending, and transformer/post-training mechanisms as a contrasting implementation for language-model blending.

In conceptual integration theory, blending is not a rare special event but a general operation of meaning construction, including ordinary language use, gesture, analogy, categorization, and framing. However, despite its ubiquity, blending is non-trivial. The present analysis emphasizes a distinction between the ubiquity of blends and the explanatory and meaning-constructive role of blending. What matters for the present argument is the provenance and functional role of models’ goal-like constraints not whether language model output can be called a blend.

Additionally, I use the term “goal” at multiple explanatory levels that should be disambiguated. At a neurocognitive level, goals refer to allostatic physiological regulation constraints that organize perception, action, and learning (Barrett and Finlay, 2018). At a behavioral level, goals are active or planned tasks or desired states that make some features of a situation relevant and others ignorable. Below, when I introduce a toy computational model of blending, goals are modeled as high-level predictions passed down a hierarchical generative model that set expectations about lower-level affordances, observations, or actions. Finally, I characterize the goal of language models as proxy-goals not because they are without goals at the representational level, but because the relevant purposiveness is supplied by training, alignment, prompt context, and decoding rather than by an embodied control system with goals ultimately grounded in maintaining its physiology.

## 2 Blending by humans and language models

I characterize human blends as generative-causal blends. On this view, human blends are constructed by generative causal models (Bottemanne, 2025) of goals and how to realize them, such as situated affordances perceived in the environment, and actions that engage the affordances to achieve the goal. In contrast, I characterize language model blends as distributionally novel blends. Language model blends are generative compositions that are novel relative to the training distribution yet consistent with learned statistics. Language models are trained to predict the next token and later shaped by alignment, e.g., via instruction tuning, direct preference optimization (DPO; Rafailov et al., 2023), and reinforcement learning from human feedback (RLHF; Ouyang et al., 2022). Language models learn blending regularities from corpora rich with blends: natural language. Apparent goal-directedness of language model blends arises from alignment and the distribution of the tokens in the context window at inference time (Vaswani et al., 2017) rather than deriving from an intrinsic causal model of a goal.

The contrast I draw is not between novelty versus non-novelty or between generative construction versus congruence with learned statistical patterns, but between the source of purposiveness underlying blend construction. Humans’ generative-causal goals construct a blend that categorizes percepts with respect to the goal, giving the percepts situated meaning. Language models exhibit extrinsic proxy-goal constraints induced by alignment and the framing of the prompt. Thus, language model novelty is real, but its goal-directedness is externalized in the alignment and prompt, rather than internalized as a causal model. Language models are generative in the sense that they model conditional structure over sequences, and they can encode abstract regularities that resemble aspects of world structure, causal relations, and perhaps goals contextualized within the training corpora. Nonetheless, in the human case, a goal is part of a control system that organizes attention, perception, and action around self-maintaining needs. In the language-model case, goal-directedness is induced by training objectives, post-training, prompt framing, context, and decoding procedures. These mechanisms can produce coherent and creative blends and exploit latent structured representations, but the goal constraint is not coupled to persistent self-maintaining needs. Thus, in what follows, “generative-causal” and “distributionally novel” should be read as general characterizations of different control architectures, not as mutually exclusive claims about whether either system contains structure.

### 2.1 Goal-directed blending

**Figure 1:**
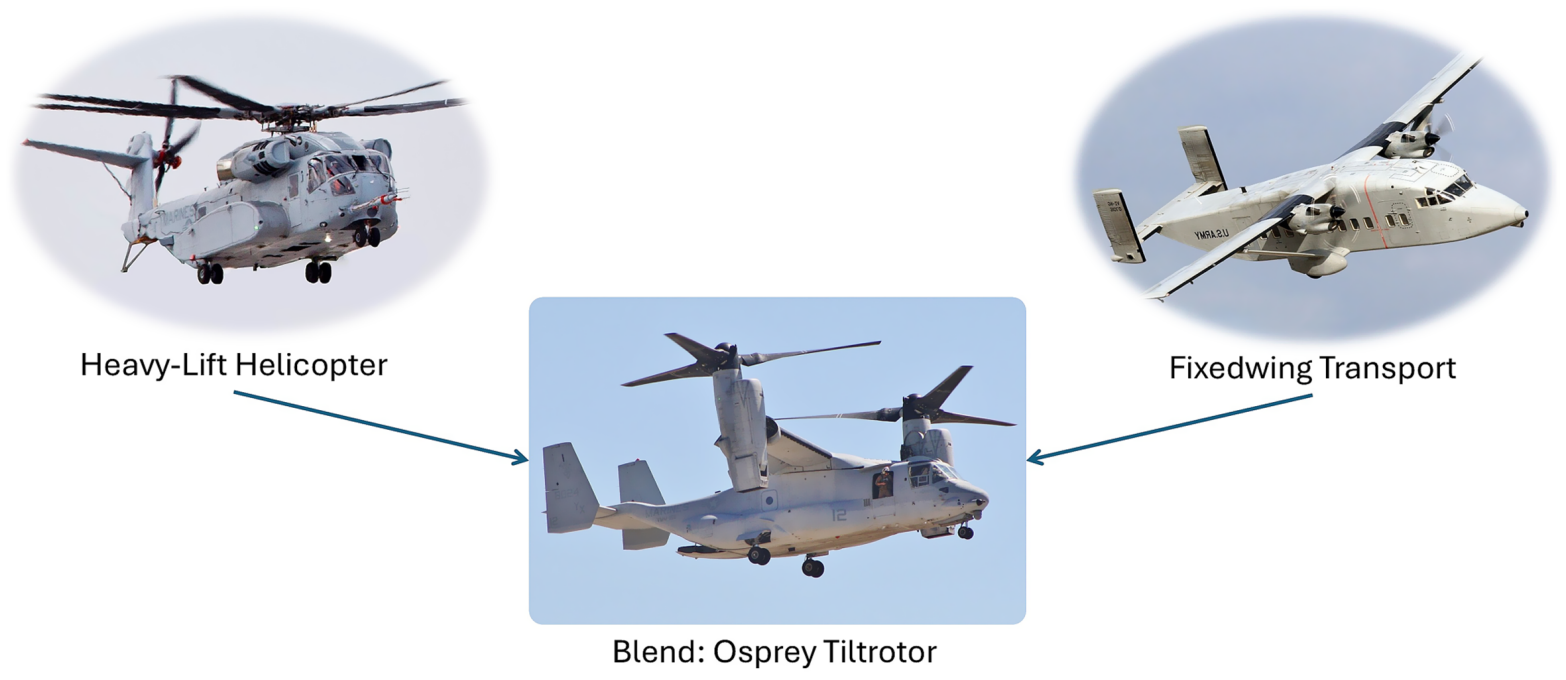
Conceptual blending in the service of reasoning about novel perceptions. Source images, from commons.wikimedia.org, are licensed under CC BY-SA or are public domain.

To structure blending, a goal foregrounds particular features of the source concepts to generate a blend that has meaning with respect to the goal (Fauconnier and Turner, 2002). For example, imagine you have never seen an Osprey tiltrotor aircraft (Figure 1). If you saw one ascending vertically, you might puzzle about the flight characteristics of this novel percept that has vertical propellers like a helicopter mounted on wings like those of an airplane (Lynn et al., 2024). You might blend your relevant prior knowledge of how helicopters and airplanes fly to infer a capability for both vertical take-off and landing (VTOL) and fast forward flight. Your goal, to understand flight characteristics of this novel contraption, leads to the construction of a model of flight characteristics that is a blend of the source concepts helicopter and airplane. The goal directs which features of the sources that are inherited by the blend: features you believe to be relevant to flight characteristics, such as orientation of propellers and presence of wings. For you, the blend is a novel concept that you use to categorize the percept. The act of categorization brings understanding; categorization applies the knowledge captured by the new concept to the percept, enabling reasoning about the percept. The blend does not need to include knowledge irrelevant to the goal, such as the number of windows and their cost because, based on your prior knowledge of flight, you believe those characteristics to be incidental to the flight characteristics.

Conventionalized blends, like “mothership” will have had goals that led to their invention and meaning but may be lost to history; they are “entrenched” (Fauconnier and Turner, 2002); no longer generative as individual lexical items. To reconstruct possible goals *post hoc* we can ask, What novel role or combination of affordances does the blend provide that neither source by itself supplies? As a blend of the sources “mothers” and “ships,” mothership, for example, is a concept that captures the idea of a larger ship sheltering, transporting, perhaps maintaining smaller ships (Hedblom, 2020); a ship that instantiates mother-related roles relative to other ships.

### 2.2 Mechanism of blending by humans

**Figure 2:**
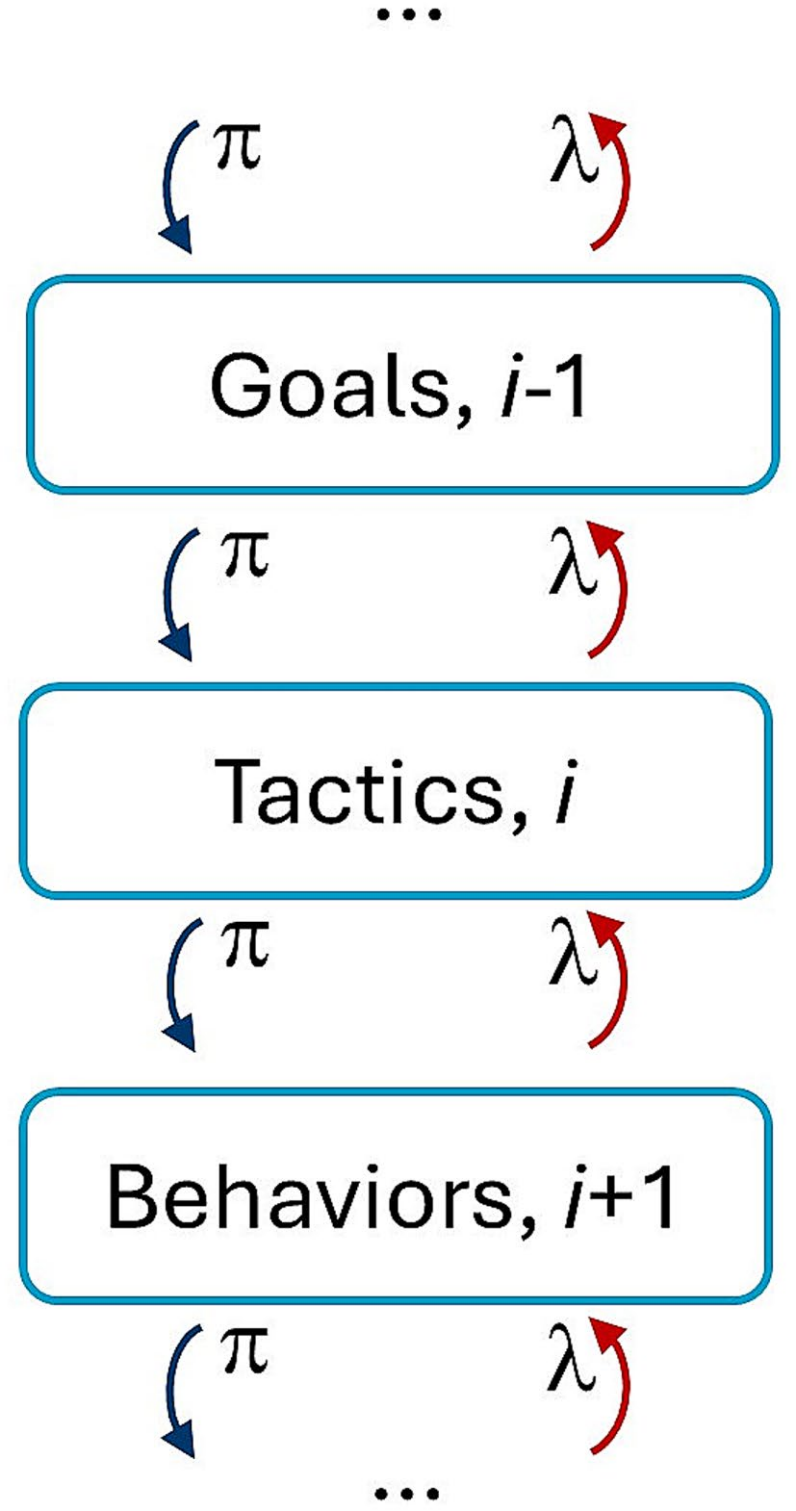
Schematic illustration of belief propagation and message passing as a model for predictive processing, across hierarchical layers, *i*, from more abstract concepts downward to more concrete concepts (adapting Pearl’s (1988) notation: prediction, *π*; prediction error, *λ*).

I model human goals as generative-causal blend constructors. A candidate mechanism that can support goal-directed blending that is consistent with neuroscience is predictive processing (Friston, 2010; Hohwy, 2013; Clark, 2016). Predictive processing is a broad and contested research program rather than a single settled theory of cognition. Here, I use it as a candidate mechanistic and computational framework for goal-directed blending. While important critiques warn against treating predictive processing as an all-purpose explanation of cognition (Litwin and Miłkowski, 2020; Williams, 2022, 2020), the architecture provides a useful formalization of how more abstract expectations can predict more concrete features, compare those predictions to observations, and update expectations or take action in response to precision-weighted error signals. Predictive processing is a hierarchical generative model of perception, latent causes, and action. A predictive processing architecture treats each layer of a hierarchical model as a generative predictor of the layer below it. For perception, higher level expectations predict lower-level features, which predict lower-level sensory inputs. For action, higher-level goals predict lower-level tactics which predict lower-level behaviors and motor actions. Predictive processing is often expressed in probabilistic generative terms (Figure 2). Such a Bayesian approach to predictive processing (e.g., Pfeffer and Lynn, 2019) provides useful, inspectable vocabulary for describing the role of goals within the formulation of embodiment described here. In Bayesian terms, predictive processing operates over probability distributions (e.g., a distribution may describe expectations about what will be seen in a given environment). The generative algorithm samples from the distribution (e.g., infers the likelihood that a category of object is present, responsible for causing a specific exemplar to be observed). In a predictive processing model, predictions are messages sent downward from more abstract parent layers (e.g., a distribution over different methods that could achieve a goal) to more concrete child layers (e.g., actions that implement a specific method). Children use the prediction as their Bayesian prior and treat it as an hypothesis (i.e., the parent’s belief about the world) that they test. Children test their parents’ predictions by comparing the prior to an observation, a message sent upward to them by their own children. Comparing the observation to the prediction yields a prediction error that the child sends upward to its parent (who receives that error message as its own observation!). Prediction errors and the precision of the priors and the observations are used to perform a Bayesian update of predictions for the next cycle. Eventually, this process leads to beliefs and actions based on the priors when the observations are relatively less precise than the priors or based on the observations when the observations are relatively more precise than the priors.

Under a predictive-processing account, blending is inference over latent conceptual structures that performs context- or goal-mediated concept construction. More specifically, blending can be modeled as precision-weighted feature selection among co-active conceptual structures. On predictive-processing accounts, a concept can be treated as a high-level structure that flexibly recruits lower-level representations depending on context (Michel, 2023, 2020). As a mechanism of blending, when two or more source concepts are active, the current goal or interpretive context raises the precision of features, roles, and affordances of those concepts that are expected to matter and lowers the precision of features that are irrelevant or misleading. The selected high-precision features are then integrated into a blended space where they are tested against observations, imagined simulations, linguistic context, or action possibilities. Prediction error is generated when the candidate blend fails to satisfy the goal, and subsequent inference revises which features are retained, suppressed, or reweighted. Blending is a reusable pattern of predictive-processing organization: a goal/context parent node with multiple source-concept nodes supplying its priors; an integrated blend-hypothesis node receiving precision-gated feature or role selections from the goal node, and which is itself a parent to observation node(s); and feedback from prediction error passed from each child to its parent(s).

**Figure 3:**
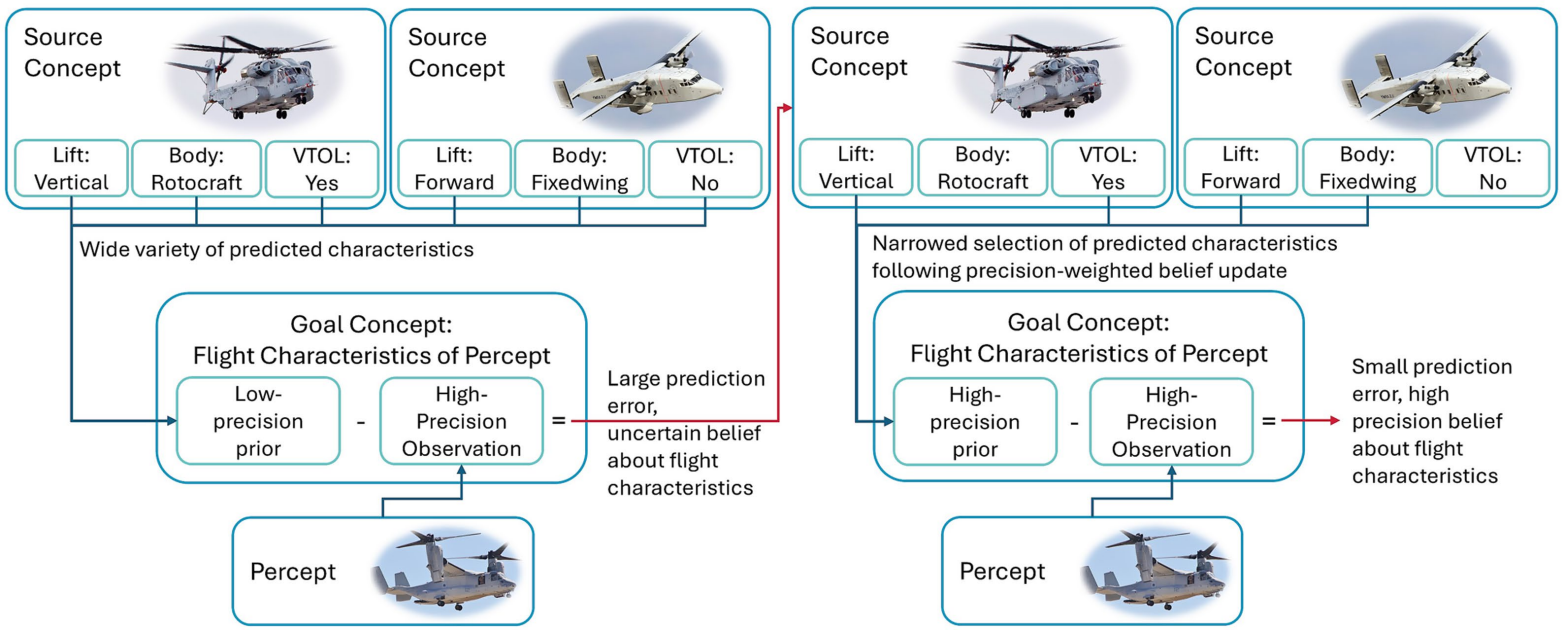
Blending as predictive processing.

In the Osprey example (Figure 3), source concepts are parent nodes that provide structured candidate features, roles, relations, and affordances. These features are predictions sent to the blend node, used as prior expectations about its observations. The goal or context functions as a control constraint that selects among possible source concept elements. In the Osprey example, an agent has the goal of understanding a percept that happens to be novel, and so the node representing the goal is the same as the node that comes to have the blended knowledge. In this example, the source concepts are the parents of the goal and the blend is the resulting hypothesis; a selectively integrated configuration of prior knowledge that predicts how the relevant features from the sources jointly satisfy the goal.

In Figure 3, the system first tries to explain the Osprey as a known aircraft, comparing features related to flight characteristics of known aircraft (i.e., affordances of flight) with observations of the Osprey. For simplicity, imagine this happening in series. First, the goal node receives predictions about how helicopters fly as its prior, compares those to the observed Osprey, calculates prediction errors, returns the errors to its helicopter parent, and the parent uses those errors to perform a precision-weighted belief update resulting in refined predictions returned to the goal node. Next follow other aircraft: fixedwing, perhaps blimps, etc. As categories of aircraft, helicopter and airplane produce moderate errors and everything else produces large errors (left side of Figure 3). However, decomposed, some helicopter and airplane features produce small prediction errors; combined, they explain some features of Osprey flight very well producing a stable blend (right side of Figure 3).

As a model of blending, predictive processing provides the grounding of goals and blends via the theory’s origins in neuroscience (Allen and Friston, 2018) and link to physiological allostasis as the root biological goal that scaffolds perception, action, and thought (Barrett and Finlay, 2018; Barrett and Simmons, 2015; Theriault et al., 2025). This account of blending does not claim that all predictive processing inferences are instances of conceptual blending or that this example of understanding novelty is the same blending circuit as that for problem solving or knowledge contextualization. Blending refers to cases in which (i) two or more structured mental spaces (i.e., concepts) must be put into correspondence and (ii) a goal or other form of context selects which correspondences are relevant. In a predictive-processing implementation, this requires a circuit in which source-concept nodes make different features available; a goal or context node sets precision over those features; a blend node integrates selected features into a candidate hypothesis; and error feedback from perception, simulation, or linguistic context provides evidence about whether the candidate blend explains the observations. Predictive processing supplies the message-passing, precision weighting, and error-revision dynamics; conceptual blending specifies the cross-space structure that those dynamics operate over.

See the Supplemental material for an implementation of the Osprey scenario in python as a toy predictive-processing-style inference using a vector symbolic architecture (VSA; Kanerva, 2009; Plate, 1995). The purpose of the toy model is to show how goal-conditioned source selection, vector-symbolic binding, precision-weighted comparison, and blend stabilization can be implemented in principle. Treat it as a model-building hypothesis. If blending is implemented in a predictive-processing architecture, then precision-weighted selection, prediction error, and hierarchical control provide plausible mechanisms for goal-conditioned feature inheritance.

### 2.3 Mechanism of blending by language models

I characterize language model blending as distributionally novel blends driven by proxy-goals. Instruction-tuning, human-preference optimization, and how the context window frames constraints at inference time all induce patterns of model response that approximate purposiveness with respect to the prompt. These are proxy-goals (Ouyang et al., 2022): exogenous supervision that contrasts with humans’ intrinsic embodied goals. Language models implement attention-based sequence modeling. Alignment methods such as RLHF and DPO steer models toward human-preferred continuations, making behavior appear goal-directed. The pretraining objective is next-token prediction; post-training reshapes the learned conditional distribution toward human-preferred responses. During inference, the context window acts as a form of working memory that conditions composition and constraint satisfaction, while in-context learning (Olsson et al., 2022), the model’s ability to adjust execution from information in the current prompt/context window (e.g., via a few examples, an instruction, or other structured cues) allows transient adaptation without weight updates. Decoding-time control further narrows the distribution of acceptable response sequence continuations (Vaswani et al., 2017). These processes combine to introduce exogenous preference signals that provide the model with goals that drive its construction of blends.

This description should not be read as a claim that language models capture only surface statistics or lack structured representations. Work on post-training and reasoning models shows that reinforcement learning and inference-time scaling incentivize stable reasoning strategies, including self-reflection, verification, and dynamic strategy adaptation (Guo et al., 2025; Kumar et al., 2025). Similarly, language models exhibit instrumental knowledge and partial, task-conditioned world models. They may infer task structure, encode latent regularities relevant to action and state transitions, and in some settings be induced or fine-tuned to perform world-model-like functions such as precondition and effect prediction (Ding et al., 2026; Gurnee and Tegmark, 2024; Xie et al., 2025; Yildirim and Paul, 2024). The contrast is that the purposiveness of a language model blend is induced by training objectives, post-training, prompt framing, context, tools, and decoding, whereas human generative-causal blending is embedded in a perception-action system with embodied goals, intervention, persistent memory, and error correction. The difference is one of source, grounding, and causal closure of the control loop, not a simple dichotomy between meaning and statistics.

**Figure 4:**
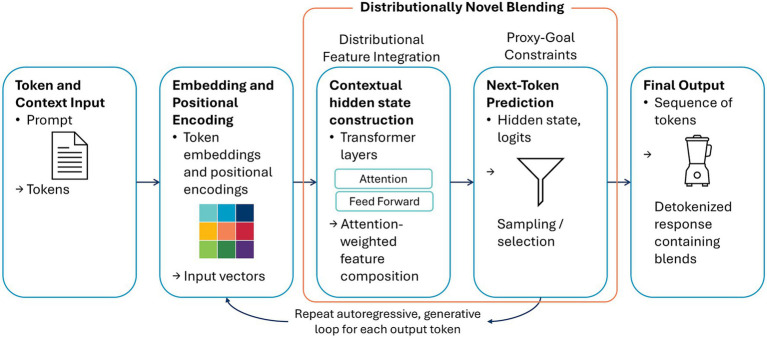
Language model processes creating distributionally novel blends.

Figure 4 schematizes how language models generate blended outputs. During tokenization, words or word fragments are mapped to learned embedding vectors and combined with positional information. Across successive transformer layers, self-attention, feed-forward transformations, residual connections, and normalization operations iteratively construct context-sensitive representations of those tokens. Concept-like structure is distributed across learned parameters and emerges dynamically from the interaction of embeddings, attention patterns, layer-wise transformations, and the current context. During decoding, the model uses these contextualized representations to produce a probability distribution over possible next tokens. Blending arises as the model composes statistically associated features, roles, and constraints into a continuation that is coherent with the prompt, training distribution, alignment history, and decoding procedure. The embedding matrix and these subsequent processes are the key elements of language model blending capability and together are an important sense in which language models have “models” or human-like concepts. These are the processes producing the fluent output, novel constructions, and sometimes insightful integration of ideas. Interestingly, entrenchment, describing blends like “mothership,” compounds and conventionalized phrases lexicalized in memory rather than constructed anew at each use or encounter (Fauconnier and Turner, 2002), has parallels with the embedding matrix. A language model does not have to reconstruct the meaning of mothership by blending “mother” with “ship” each time, yet as a token, “mothership” is still identifiable as a blend via its distributional embedding geometry and contextual activation patterns.

## 3 Embodiment and conceptual flexibility

While there are clear architectural differences that distinguish blending by humans and language models, those differences ultimately center on embodiment and its deep role in how brains understand perceptions. On the embodied view, one’s goals constrain how one categorizes (i.e., understands) the environment. For example, if you need to change a lightbulb, you can perceive a “chair” as a “stepstool”; the goal leads to recognizing stepstool-like affordances in the chair percept (Lynn et al., 2023). The ability to achieve the goal requires (a) framing the goal as a problem whose solution is a set of recognizable affordances (e.g., “elevation,” “support”), (b) decomposing percepts into features that manifest those affordances, and (c) blending the features into a unified concept that you can act on (reaching the bulb). The flexibility of human goals, affordances, and blending are intrinsic to the body’s morphology, physiology, and sensory and motor capabilities as instantiated and simulated in its predictive, causal architecture. An embodied and predictive view, then, highlights three distinctions between distributionally novel blends and human generative-causal blends that are salient with respect to understanding the relevance of language model proxy goals versus human intrinsically embodied goals on conceptual flexibility.

The first distinction has to do with the nature and use of affordances. On an embodied cognition view, affordances are situated, goal-relative applications of “pre-conceptual” constructs that structure perceived relationships between entities in the environment. Known as image schemas (Lakoff and Johnson, 1980) and force dynamics (Talmy, 1988), these constructs function as a kind of grammar or logic (Hedblom et al., 2025); a domain-specific language of conceptual structure. Patterns such as containment, support, source-path-goal, blockage, enablement, resistance, linkage, and balance provide combinable, reusable structure for interpreting situations and constructing blends. Image schemas and force dynamics are recognized in the environment and blended as abstract solutions to attaining goals (Hedblom, 2020). Affordances are situated, goal-relative applications of those structures to perception and action: a chair can be understood not only as a chair, but as elevation, obstacle, container-for-objects, or barricade depending on the active goals.

Language models acquire linguistic traces of these image-schematic and force-dynamic regularities. What they lack is not necessarily all access to the structures, but the perception-action grounding that makes those structures operative constraints for intervention, error correction, and goal satisfaction. Thus, an LLM’s suggestion to, e.g., use a chair as a stepstool, is a linguistic substitution constrained by distributional statistics and alignment, not a sensorimotor commitment that reorganizes perception/action policies, and this distinction limits language model flexibility in novel situations.

The second distinction is about superposition vs. the conceptual structure provided by affordances. Language model blends arise through attention-weighted superposition in high-dimensional spaces, where multiple features share vector directions. This architecture supports high feature capacity and yields coherent blends when training priors support them, but also produces interference during decoding if constraints conflict (Elhage et al., 2022). Under such intersecting constraints, tokens can be selected from regions lexically consistent with all constraints while not satisfactorily addressing any, yielding responses to prompts that feel like generic compromises of goals. In contrast, on the predictive processing view, image schematic and force-dynamic affordances occupy middle layers of human hierarchical causal models, between sensor inputs and concepts, providing distinct structured constraints, reducing interference, or resolving it with error-driven refinement during blend generation.

The third distinction is that the generativity of language models is not the same as that of brains. Language models can approximate Bayesian updating via in-context learning (Xie et al., 2021) and additional mechanisms (e.g., subsequent prompt refinement, alignment, external agentic tooling), but the base “goal” is still generation of token-likelihood under correlational regularities from pretraining and contextualized by the prompt, rather than causal hypothesis generation to explain observations (Wu et al., 2024). Thus, language models show short term adaptability but not lasting model change or explicit causal roll-out, whereas humans’ model revision and counterfactual testing support revision and generalization after errors or novelty. Additionally, predictive-processing accounts treat precision of both priors and observations as a controllable variable (Friston, 2010), providing meta-cognitive monitoring and reallocation of attention under uncertainty. Language models emulate reflective behavior via prompted protocols (e.g., chain-of-thought, self-consistency checks) and alignment, but these are heuristics without intrinsic uncertainty-aware controllers such as belief precision or error assignment across model layers or factors. Humans detect and repair incoherence proactively; language models need scaffolding to do so and are brittle when reflection is not prompted or conflicts with prior alignment.

Differences in generativity are of organization and function rather than an all-or-none opposition. Multimodal models, tool-using agents, and robot policies can acquire forms of grounding, and post-training can make their behavior more coherent and strategically organized. Work comparing language model and human concept norms suggests that ungrounded language models can recover non-sensorimotor conceptual structure while diverging more strongly from human representations in sensory and motor domains (Xu et al., 2025). The difference is most consequential when conceptual blending must reorganize perception and action, less when the blend is a familiar linguistic or high-level relational pattern. The distinction highlighted here is whether the system’s blend is anchored in a closed perception-action loop in which goals specify required affordances, action tests those affordances, and prediction error revises the model over time. Human conceptual flexibility depends on that loop.

These three distinctions explain why language model blends can look agile in well-prompted contexts yet manifest brittleness in the open-ended settings that represent the core strengths of human conceptual flexibility, such as settings requiring long-horizon goals, causal affordances, interference resolution, and persistent model change (Pezzulo et al., 2024). The distinctions suggest that the next step is not simply larger models, longer contexts, or more elaborate prompting, but architectures that make affordance recognition an internal computational target. An AI that reasons more like an embodied blender would not begin with “what token comes next?” but with “what structure would satisfy this goal?” Such a system would translate goals into required affordances, represent those affordances using reusable image-schematic and force-dynamic structures, search perception, memory, simulation, and tools for candidate realizations, and then revise the candidate blend through prediction error. In this architecture, language remains useful as an interface, as it is between humans, but the reasoning chain is goal to affordance specification to candidate realization to causal test to revision (Pezzulo et al., 2024). This would move AI blending from fluent composition toward functional construction: the ability to infer that a novel tiltrotor percept can be conceptualized as a helicopter-airplane blend for flight reasoning or an object can satisfy a goal because it instantiates the right relational structure, even when no familiar label is available.

## 4 Conclusion

While computational blending is often discussed in the context of creativity (Confalonieri et al., 2018), the contrasts I describe between human and language model blending are not about novelty versus non-novelty, but between the mechanism and function of goals. Both humans and language models generate novel blends, but they do so with different mechanisms and with potentially different functional consequences. I have argued that humans produce generative-causal blends that can be modeled with a predictive processing architecture that furnishes a hierarchical, generative account of perception and action. The goal causing the blend is not merely an externally applied instruction; it is an active parent node within the generative hierarchy that predicts the affordances and mid-level structures its children should test against observations. The goal modulates priors and precision, sends messages downward to recruit image-schematic/force-dynamic constraints, and receives prediction errors upward to refine or repair the blend. By contrast, language models produce distributionally novel blends for which the goal is a proxy signal that steers token probabilities via self-attention and alignment and decoding heuristics. The goal-directedness is externalized, residing in the context window, the instructions, and human preferences, rather than in an internal causal model. Proxy-goals keep constraints salient short-term, but remain outside the model’s generative mechanism, more like working-memory than a revisable, hierarchical controller. Proxy goals do not encode explanations of why a particular blend should exist or how it would work.

The distinctions between distributionally novel blends and human generative-causal blends intersects somewhat with debates about notions of grounding (Mollo and Millière, 2026) and embodiment (Wilson, 2002), and whether language model output can have meaning other than that attributed to it by human users. Some accounts argue that language models can acquire critical features of conceptual competence or congruence with how humans describe the world from their training and post-training (Ma and Narayanan, 2026; Mollo and Millière, 2026; Xu et al., 2025); others argue that the lack of stable sensorimotor grounding imposes limits (Bender and Koller, 2020; Pavlick, 2023; Shanahan, 2023). With respect to this debate, for humans, the goals that direct blending and the concepts that are blended are embodied, by which I mean: Goals are ultimately hierarchically rooted in physiology, categorization of percepts is with respect to those goals (Barrett and Miller, 2026), and the affordances required to achieve a goal are shaped by sensory/motor capabilities or are themselves other embodied concepts. Thus, for brains, embodiment is the medium of conceptual flexibility and grounds our semantics as contextualized by situated goals. For language models, it could be noted that a prompt is a percept, patterns learned about how to generate outputs aligned with proxy goals are affordances, and the response to the prompt is an action expressing goal-directed blends. On this view, what the model might be said to meaningfully understand is a latent causal relationship between the prompt and the response because the prompt and the response are referentially grounded through training that co-selects them (Mollo and Millière, 2026). This causal model is “embodied” in the model architecture and its training experience. However, that grounding may not be the same as having a common basis of understanding with the human about the contents of the prompt or the interpretation of the response.

For many current use cases, the capability for distributional novel blends exhibited by language models is of widespread practical use. However, the distinctions between human and language model blending reflect evolutionary, developmental, and biological processes that enable autonomy and conceptual flexibility, with implications for autonomous general AI and robotics. A practical research program follows from these distinctions. Future AI systems should be trained to construct and test blends as goal-satisfying affordance models. One path is to add an intermediate affordance layer between linguistic goals and low-level perception/action, a “mental space” (Fauconnier and Turner, 2002) in which goals are represented as image-schematic and force-dynamic requirements. Training would then focus on identifying objects, actions, and plans that realize those requirements across changing contexts. Such a regime would require counterfactual simulation, intervention, multimodal grounding, persistent memory, and an organizing principle to teach the agent to ask what affordances the goal requires. If successful, this would turn conceptual blending from an expressive talent into an architectural capability for adaptive reasoning. The relevant advance would be a system that reasons through roles and derives the meaning of objects, plans, and concepts via the affordance structures they can instantiate relative to a goal, using blending to achieve the human-like conceptual flexibility.
