# Supplementary material for: Conceptual blending in humans and language models
Source: Front Psychol. 2026 Jul 29;17:1849083. doi: 10.3389/fpsyg.2026.1849083 (PMC13461484; doi:10.3389/fpsyg.2026.1849083)
Supplement: Supplementary file 1 [file Supplementary_file_1.DOCX]

Supplementary Material

This supplementary material illustrates an implementation of the Osprey scenario from the main text in python as a toy predictive-processing-style inference using a vector symbolic architecture (VSA) representation.

# 1 Scenario Summary

The main text describes goal-directed blending as the construction of a novel concept from multiple source concepts, where the selection and integration of elements from the sources are guided and constrained by the affordances required to satisfy an active goal. I use predictive processing (Friston 2010) as a biologically inspired computational implementation of blending. To illustrate goal-directed blending in a predictive processing framework, imagine the following scenario: An agent observes an unfamiliar aircraft (an Osprey) ascending vertically, spawning a goal to understand the aircraft’s flight characteristics. The agent’s prior knowledge includes several flight classes and the tradeoffs associated with each. Because the object’s vertical lift capability resembles a helicopter while its morphology suggests fixed-wing cruise capability, no single known category adequately explains the observation; as priors for the conceptual node representing the novel observation, all lead that node to generate substantial prediction error compared it its observation of the object. To reduce prediction error and support future behavioral predictions, the agent constructs a blended latent hypothesis: a hybrid aircraft combining rotor-based vertical take-off and landing (VTOL) with wing-based efficient forward flight. Predictive processing maps latent causes (goals, affordances, perceived structure within a scene) to predicted sensory states. Blending selectively recruits and integrates helicopter-like and airplane-like structures into a new blended conceptual model whose organization is governed by functional compatibility rather than superficial similarity, with the aim of explaining the surprising observation. From the blend, the agent could go on to infer likely hover capability, improved cruise efficiency relative to helicopters, a regime-transition mechanism, and associated complexity/maneuverability tradeoffs.

# 2 Modeling Approach

A minimal computational demonstration shows how a predictive-processing-style inference routine can be implemented in a VSA (Kanerva 2009; Plate 1995) to model goal-directed blending. The example is deliberately small so that the relationship between the conceptual claims and the code remains easy to inspect. I illustrate predictive processing in a VSA because it encodes, composes, and decomposes hierarchically structured symbolic information as distributed vectors, enabling the predictive processing machinery to perform generative causal reasoning directly on the architecture (Furlong and Eliasmith 2023) while maintaining human inspectabiliy. VSA combines symbolic expressiveness with statistical resilience, creating a computational substrate for symbolic-subsymbolic AI and cognitive architectures.

In VSA terms, stored aircraft schemas are encoded as weighted bundles of role-filler bindings (e.g., LIFT_MODE:VERTICAL, BODY_FORM:FIXED_WING). An unfamiliar aircraft observation is encoded in the same form. Each stored schema serves as a predicted observation under an associated hypothesis, and similarity to the actual observation is computed by cosine similarity in the VSA space. Prediction error is defined as one minus cosine similarity. Posterior support for each hypothesis is then computed from the prior and an error penalty term. To illustrate generative blending, the top two hypotheses are superposed to form a hybrid candidate, which is evaluated against the same observation. In the scenario, the observation combines vertical rotor-based lift with fixed-wing body structure, so no single stored schema fits optimally. A helicopter-airplane blend reduces error more effectively, demonstrating how a VSA representation can support structured predictive comparison and provisional hybrid explanation.

In blending terms, the inferential goal constrains what counts as useful conceptual structure. The model is not blending aircraft types arbitrarily. Rather, it blends hypotheses because a mixed explanation better accounts for the observed conjunction of vertical lift behavior, rotor-based propulsion, and fixed-wing body form. In this sense, the blend is not an unconstrained average based on perceived similarity. It is a response to residual mismatch under single-source explanations. The hybrid emerges because it provides a better explanatory organization for the observed feature bundle.

In predictive processing terms, the agent’s higher-level flight-schema generate predictions for different aircraft types for the node representing the new concept to receive as priors and compare to its observations. Observational evidence coming into that node fits parts of both helicopter-like and airplane-like models but is not well explained by either prediction alone. This persistent prediction error triggers an abductive update: a new composite latent hypothesis is formed that inherits rotor-based vertical lift expectations from one source model and efficient forward-flight expectations from another. This blended hypothesis reduces prediction error, offering a better explanation of the observations.

The objective of this implementation is not to provide a full predictive processing model, but to demonstrate in a transparent way how predictive processing can model goal-directed, generative concept blending. Relevant ingredients of predictive processing implanted here in VSA include:

- Structured priors over candidate hypotheses. The stored aircraft categories and their initial prior probabilities correspond to the agent’s initial expectations.
- Top-down predictions generated from priors. Each stored prototype serves as a top-down prediction of what would be observed if that hypothesis were true.
- Prediction error. Mismatch between a prediction and an observation.
- Precision weighting of prediction error. Precision enters as weights used when building the observation bundle. Features with greater observational confidence contribute more strongly to the vector that drives the comparison. Thus, some discrepancies matter more than others because some observed features are treated as more reliable.
- Belief updating from priors to posteriors. Posterior support is obtained by combining prior plausibility with precision-weighted prediction error. Hypotheses that better reduce prediction error receive increased posterior support.
- Blended hypothesis generation. When no single prior sufficiently explains the observation, a blended candidate can further reduce error. In this toy setting, blending is operationalized as vector superposition. Conceptually, this corresponds to introducing a new composite hypothesis that inherits explanatory structure from more than one familiar source.

# 3 Python Code

"""

Demonstration of goal-directed blending using Vector Symbolic Architectures (VSA) to implement a Predictive Processing (PP)-style inference to motivate and execute the blend.

© 2026 Charles River Analytics, a GRVTY Company

Spencer Lynn, slynn@cra.com

www.cra.com

**SCENARIO**

--------

An agent observes an unfamiliar aircraft (an Osprey). The observation is:

    - it rises vertically

    - it appears to use rotor-like propulsion

    - it has a fixed-wing-like body

    - it is not buoyant like a blimp

    - it is not flapping like an ornithopter

The agent does not already know the category "tiltrotor" or "Osprey".

It does, however, know some familiar flying things:

    - airplane

    - helicopter

    - blimp

    - ornithopter

Each known aircraft type is represented as a prototypical structured set of expected features.

The script does the following:

    1. Build VSA symbol vectors for roles and fillers.

    2. Build structured prototype vectors for each aircraft type.

    3. Build a structured observation vector for the unfamiliar craft.

    4. Compare each prototype prediction to the observation.

    5. Compute a precision-weighted prediction error.

    6. Update beliefs from prior to posterior.

    7. Test whether blending the best two hypotheses improves the explanation.

This is not intended to be a full mathematically faithful implementation of all predictive processing details. It is a toy script meant to be readable and illustrative. The comments are intentionally very detailed, so that readers unfamiliar with Python, Predictive Processing, or VSA can learn by reading the file.

"""

**# ============================================================================**

**# IMPORTS**

**# ============================================================================**

\# We need NumPy for vector math.

import numpy as np

**# ============================================================================**

**# RANDOM SEED**

**# ============================================================================**

\# Setting a random seed makes the script reproducible. If we run it again with the same seed, you get the same random vectors and the same numerical results.

np.random.seed(13)

**# ============================================================================**

**# BASIC VSA UTILITIES**

**# ============================================================================**

\# In a Vector Symbolic Architecture, symbols are represented as high-dimensional vectors. Structured information is built by:

\#   - creating atomic vectors for symbols

\#   - binding roles to fillers of those roles

\#   - bundling multiple bound pairs together

\#

\# Here we will use:

\#   - random bipolar vectors for atomic symbols

\#   - elementwise multiplication for binding

\#   - vector addition (superposition) for bundling

\#   - cosine similarity for comparing vectors

**def random_bipolar_vector(dim):**

    """

    Create a random bipolar vector of length 'dim'.

    Why bipolar?

    ------------

    Bipolar vectors are useful when you need a balance between computational efficiency, high robustness to noise, and straightforward similarity metrics.

    Parameters

    ----------

    dim : int

        Dimensionality of the vector space.

    Returns

    -------

    np.ndarray

        A 1D NumPy array of shape (dim,) with values +1 or -1.

    """

    return np.random.choice([-1.0, 1.0], size=dim)

**def normalize(vec):**

    """

    Normalize a vector to unit length. If the vector somehow has zero length, return it unchanged.

    Why normalize?

    --------------

    When comparing vectors using cosine similarity, it is often helpful to keep vector magnitudes under control. Unit-length vectors make interpretations more stable.

    Parameters

    ----------

    vec : np.ndarray

        Input vector.

    Returns

    -------

    np.ndarray

        Unit-length version of the vector.

    """

    norm = np.linalg.norm(vec)

    if norm == 0:

        return vec

    return vec / norm

**def bind(a, b):**

    """

    Bind two vectors together using elementwise multiplication.

    In VSA, binding is used to represent a relation such as:

        ROLE : FILLER

    Example:

        bind(LIFT_MODE, VERTICAL)

    The result is a new vector representing the structured pair.

    Why elementwise multiplication?

    -------------------------------

    It is a standard and simple binding operation in several VSA families. It is easy to implement and works well for toy demonstrations.

    Parameters

    ----------

    a, b : np.ndarray

        Vectors of the same shape.

    Returns

    -------

    np.ndarray

        The bound vector.

    """

    return a * b

**def bundle(vectors, weights=None):**

    """

    Bundle (superpose) a list of vectors into one vector.

    Bundling is how we combine several pieces of information into a single distributed representation.

    Example:

        aircraft_vector = bundle([

            bind(LIFT_MODE, VERTICAL),

            bind(PROPULSION, ROTOR),

            bind(BODY_FORM, FIXED_WING)

        ])

    If weights are supplied, each vector is multiplied by its weight before being added. This is a convenient way to mock-up precision weighting for a toy example.

    Parameters

    ----------

    vectors : list of np.ndarray

        Vectors to add together.

    weights : list of float, optional

        Weights for each vector. If None, all weights are treated as 1.

    Returns

    -------

    np.ndarray

        The normalized bundled vector.

    """

    if len(vectors) == 0:

        raise ValueError("bundle() received an empty list of vectors.")

    if weights is None:

        weights = [1.0] * len(vectors)

    # Start from a zero vector of the same shape as the first input vector.

    total = np.zeros_like(vectors[0])

    # Add each vector scaled by its weight.

    for vec, w in zip(vectors, weights):

        total += w * vec

    # Normalize the final sum so that magnitudes remain comparable.

    return normalize(total)

**def cosine_similarity(a, b):**

    """

    Compute cosine similarity between two vectors.

    Cosine similarity measures how aligned two vectors are.

        +1  means identical direction

         0  means roughly unrelated / orthogonal

        -1  means opposite direction

    In this script, cosine similarity is part of the calculation for how well a prediction matches an observation.

    Parameters

    ----------

    a, b : np.ndarray

    Returns

    -------

    float

        Cosine similarity.

    """

    a_n = normalize(a)

    b_n = normalize(b)

    return float(np.dot(a_n, b_n))

**# ============================================================================**

**# SYMBOL TABLE**

**# ============================================================================**

\# We now create atomic vectors for all the symbolic roles and fillers we need.

\#

\# ROLES are things like:

\#   - LIFT_MODE

\#   - PROPULSION

\#   - BODY_FORM

\#

\# FILLERS are the values those roles can take, like:

\#   - VERTICAL

\#   - ROTOR

\#   - FIXED_WING

\#

\# A structured feature such as:

\#   LIFT_MODE : VERTICAL

\# becomes:

\#   bind(symbols["LIFT_MODE"], symbols["VERTICAL"])

**def make_symbol_table(dim):**

    """

    Create a dictionary mapping symbol names to random VSA vectors.

    Parameters

    ----------

    dim : int

        Dimensionality of the vector space.

    Returns

    -------

    dict

        Mapping from string name to vector.

    """

    symbol_names = [

        # Roles

        "LIFT_MODE",

        "PROPULSION",

        "BODY_FORM",

        "VTOL",

        "HOVER",

        "CRUISE_EFF",

        "MANEUVER",

        "BUOYANCY",

        "FLAPPING",

        # Fillers / values

        "VERTICAL",

        "FORWARD",

        "ROTOR",

        "JET",

        "PROPELLER",

        "FIXED_WING",

        "ROTORCRAFT",

        "AIRSHIP",

        "FLAPPING_WING",

        "YES",

        "NO",

        "HIGH",

        "MEDIUM",

        "LOW",

    ]

    symbols = {}

    for name in symbol_names:

        symbols[name] = random_bipolar_vector(dim)

    return symbols

**# ============================================================================**

**# FEATURE AND BUNDLE HELPERS**

**# ============================================================================**

\# These helper functions make the script easier to read.

**def make_feature(role, filler, symbols):**

    """

    Turn a role-filler pair into one bound VSA vector.

    Example:

        make_feature("LIFT_MODE", "VERTICAL", symbols)

    Parameters

    ----------

    role : str

    filler : str

    symbols : dict

    Returns

    -------

    np.ndarray

        Bound role-filler vector.

    """

    return bind(symbols[role], symbols[filler])

**def make_structured_bundle(feature_specs, symbols):**

    """

    Build a bundled vector from a list of feature specifications.

    Each feature spec is a tuple:

        (role_name, filler_name, weight)

    The weight lets us express precision or centrality.

    Example:

        [

            ("LIFT_MODE", "VERTICAL", 1.0),

            ("PROPULSION", "ROTOR", 0.9),

            ("BODY_FORM", "FIXED_WING", 1.0),

        ]

    Parameters

    ----------

    feature_specs : list of tuples

        Each tuple is (role, filler, weight)

    symbols : dict

        Symbol table.

    Returns

    -------

    np.ndarray

        A bundled vector representing the structured set of features.

    """

    vectors = []

    weights = []

    for role, filler, weight in feature_specs:

        vectors.append(make_feature(role, filler, symbols))

        weights.append(weight)

    return bundle(vectors, weights=weights)

**# ============================================================================**

**# PROTOTYPE KNOWLEDGE**

**# ============================================================================**

\# Here we encode the agent's prior knowledge of known aircraft types.

\# Each known aircraft type is represented as a structured prototype: what the agent expects to observe if that hypothesis were true. The weights here can be interpreted as how central each feature is to that prototype. They are not probabilities. They simply scale the feature's contribution to the prototype vector.

**def build_aircraft_prototypes(symbols):**

    """

    Create VSA prototype vectors for known aircraft categories.

    Returns

    -------

    dict

        Mapping from hypothesis name to a dict with:

            - "features": the human-readable feature list

            - "vector": the VSA prototype vector

    """

    prototypes = {}

    # ----------------------------------------------------------------------

    # AIRPLANE

    # ----------------------------------------------------------------------

    # The prototype expectations here are:

    #   - not VTOL

    #   - not hover-capable

    #   - strong cruise efficiency

    #   - fixed-wing body

    #   - lift via forward-flight

    #   - jet propulsion

    airplane_features = [

        ("LIFT_MODE", "FORWARD", 1.0),

        ("PROPULSION", "JET", 0.8),

        ("BODY_FORM", "FIXED_WING", 1.0),

        ("VTOL", "NO", 1.0),

        ("HOVER", "NO", 1.0),

        ("CRUISE_EFF", "HIGH", 1.0),

        ("MANEUVER", "MEDIUM", 0.7),

        ("BUOYANCY", "NO", 0.9),

        ("FLAPPING", "NO", 0.9),

    ]

    prototypes["airplane"] = {

        "features": airplane_features,

        "vector": make_structured_bundle(airplane_features, symbols),

    }

    # ----------------------------------------------------------------------

    # HELICOPTER

    # ----------------------------------------------------------------------

    # This prototype emphasizes:

    #   - vertical lift

    #   - rotor propulsion

    #   - VTOL

    #   - hovering

    #   - rotorcraft body form

    #

    helicopter_features = [

        ("LIFT_MODE", "VERTICAL", 1.0),

        ("PROPULSION", "ROTOR", 1.0),

        ("BODY_FORM", "ROTORCRAFT", 1.0),

        ("VTOL", "YES", 1.0),

        ("HOVER", "YES", 1.0),

        ("CRUISE_EFF", "MEDIUM", 0.8),

        ("MANEUVER", "HIGH", 0.9),

        ("BUOYANCY", "NO", 0.9),

        ("FLAPPING", "NO", 0.9),

    ]

    prototypes["helicopter"] = {

        "features": helicopter_features,

        "vector": make_structured_bundle(helicopter_features, symbols),

    }

    # ----------------------------------------------------------------------

    # BLIMP

    # ----------------------------------------------------------------------

    # A blimp can rise or remain aloft by buoyancy. It can have some directional flight capacity, but its main mismatch

    # with the observed craft will be the strong buoyancy expectation and lack of rotor-based powered lift.

    blimp_features = [

        ("LIFT_MODE", "VERTICAL", 0.7),

        ("PROPULSION", "PROPELLER", 0.6),

        ("BODY_FORM", "AIRSHIP", 1.0),

        ("VTOL", "YES", 0.8),

        ("HOVER", "YES", 0.7),

        ("CRUISE_EFF", "LOW", 0.8),

        ("MANEUVER", "LOW", 0.8),

        ("BUOYANCY", "YES", 1.0),

        ("FLAPPING", "NO", 0.9),

    ]

    prototypes["blimp"] = {

        "features": blimp_features,

        "vector": make_structured_bundle(blimp_features, symbols),

    }

    # ----------------------------------------------------------------------

    # ORNITHOPTER

    # ----------------------------------------------------------------------

    # The key distinctive feature here is flapping-wing flight.

    ornithopter_features = [

        ("LIFT_MODE", "FORWARD", 0.8),

        ("PROPULSION", "PROPELLER", 0.3),  # weak / secondary here in toy setup

        ("BODY_FORM", "FLAPPING_WING", 1.0),

        ("VTOL", "NO", 0.7),

        ("HOVER", "NO", 0.8),

        ("CRUISE_EFF", "MEDIUM", 0.6),

        ("MANEUVER", "HIGH", 0.7),

        ("BUOYANCY", "NO", 0.9),

        ("FLAPPING", "YES", 1.0),

    ]

    prototypes["ornithopter"] = {

        "features": ornithopter_features,

        "vector": make_structured_bundle(ornithopter_features, symbols),

    }

    return prototypes

**# ============================================================================**

**# OBSERVATION**

**# ============================================================================**

\# Define the observation of the unfamiliar aircraft.

\#

\# The object appears to combine:

\#   - vertical lift

\#   - rotor-like propulsion

\#   - fixed-wing body

\#

\# This creates a mismatch for stored categories:

\#   - helicopter explains vertical + rotor well, but not fixed-wing body

\#   - airplane explains fixed-wing body well, but not vertical lift

**def build_observation(symbols):**

    """

    Build the observation vector for the unfamiliar aircraft.

    Here the weights can be interpreted as observation precision:

    how reliable or influential each observed feature is.

    Higher weight = higher precision = more influence on the comparison.

    Returns

    -------

    dict

        Contains:

            - "features": human-readable feature specs

            - "vector": VSA observation vector

    """

    observation_features = [

        # high confidence: we clearly see vertical rise

        ("LIFT_MODE", "VERTICAL", 1.0),

        # high confidence: rotor-like lift source

        ("PROPULSION", "ROTOR", 0.95),

        ("BODY_FORM", "FIXED_WING", 1.0),  # high confidence: wing-like body

        # high confidence: not floating like a blimp

        ("BUOYANCY", "NO", 1.0),

        ("FLAPPING", "NO", 0.9),          # fairly high confidence: not flapping

    ]

    return {

        "features": observation_features,

        "vector": make_structured_bundle(observation_features, symbols),

    }

**# ============================================================================**

**# PREDICTIVE PROCESSING-STYLE SCORING**

**# ============================================================================**

\# Implement a PP-like update.

**def compute_prediction_error(predicted_vec, observed_vec):**

    """

    Compute a simple prediction error based on cosine similarity as:

        error = 1 - cosine_similarity

    If similarity is high, error is low. If similarity is low, error is high.

    Since cosine similarity can in principle go below 0, the error can go

    above 1. In practice for these bundled representations it will usually

    stay in a manageable range.

    Parameters

    ----------

    predicted_vec : np.ndarray

    observed_vec : np.ndarray

    Returns

    -------

    tuple

        (similarity, error)

    """

    sim = cosine_similarity(predicted_vec, observed_vec)

    err = 1.0 - sim

    return sim, err

**def posterior_update(priors, errors, beta=4.0):**

    """

    Convert priors and errors into posterior probabilities.

    We use a simple softmax-like form:

        score_h = log(prior_h) - beta * error_h

    Then:

        posterior_h = exp(score_h) / sum_j exp(score_j)

    Intuition

    ---------

    - Larger prior helps a hypothesis.

    - Larger error hurts a hypothesis.

    - beta controls how strongly error matters.

    This is a simple stand-in for "belief update under prediction error".

    Parameters

    ----------

    priors : dict

        Mapping hypothesis -> prior probability.

    errors : dict

        Mapping hypothesis -> prediction error.

    beta : float

        Strength of error penalty.

    Returns

    -------

    dict

        Mapping hypothesis -> posterior probability.

    """

    scores = {}

    for h, prior in priors.items():

        # Use log(prior) so probabilities combine additively with the error term.

        scores[h] = np.log(prior) - beta * errors[h]

    # Numerical stabilization: subtract the max score before exponentiating.

    max_score = max(scores.values())

    exp_scores = {}

    for h, score in scores.items():

        exp_scores[h] = np.exp(score - max_score)

    total = sum(exp_scores.values())

    posteriors = {}

    for h, exp_score in exp_scores.items():

        posteriors[h] = exp_score / total

    return posteriors

**# ============================================================================**

**# BLENDING of ROLE-FILLER "AFFORDANCES" FROM DIFFERENT PROTOTYPES**

**# ============================================================================**

**def to_role_map(feature_specs):**

    """

    Convert [(role, filler, weight), ...] into {role: (filler, weight)}.

    """

    m = {}

    for r, f, w in feature_specs:

        m[r] = (f, w)

    return m

**def affordance_blend(**

    proto_a, proto_b, observation, symbols,

    alpha=0.5,                 # belief bias: trust in A vs B

    obs_weight=1.0,            # observation guidance only

    selective=True,            # drop roles that hurt fit

    min_role_score=0.0         # threshold for inclusion

):

    """

    Blend by selecting per-role fillers based on observation fit + mild prior bias.

    No synergy terms and no goals.

    proto_a/proto_b: dicts as in prototypes[...] with "features" and "vector".

    observation: dict with "features" and "vector".

    """

    role_a = to_role_map(proto_a["features"])

    role_b = to_role_map(proto_b["features"])

    obs_vec = observation["vector"]

    roles = set(role_a.keys()) | set(role_b.keys())

    selected = []  # [(role, filler, weight), ...]

    # Role-wise selection (observation + mild prior bias)

    for role in roles:

        cand_a = role_a.get(role, None)

        cand_b = role_b.get(role, None)

**def score_candidate(cand, prior_weight):**

            if cand is None:

                return -np.inf, None, 0.0

            filler, w = cand

            fv = make_feature(role, filler, symbols)

            sim_obs = cosine_similarity(fv, obs_vec)

            score = obs_weight * sim_obs + prior_weight

            return score, fv, w

        score_a, fv_a, w_a = score_candidate(cand_a, alpha)

        score_b, fv_b, w_b = score_candidate(cand_b, (1.0 - alpha))

        if score_a >= score_b:

            chosen = cand_a

            chosen_score = score_a

        else:

            chosen = cand_b

            chosen_score = score_b

        if chosen is not None:

            filler, w = chosen

            if (not selective) or (chosen_score >= min_role_score):

                # Keep weight near original centrality, but nudge by fit

                weight = max(0.05, w) * (0.5 + 0.5 * max(0.0, chosen_score))

                selected.append((role, filler, weight))

    # Base blended representation from selected role–filler pairs

    base_vec = make_structured_bundle(

        selected, symbols) if selected else np.zeros_like(obs_vec)

    return base_vec, selected

**# ============================================================================**

**# PRINT HELPERS**

**# ============================================================================**

\# These make the output easier to read.

**def print_feature_list(title, feature_specs):**

    """

    Pretty-print a list of (role, filler, weight) triples.

    """

    print(title)

    for role, filler, weight in feature_specs:

        print(f"  - {role:12s} : {filler:13s}  weight={weight:.2f}")

    print()

**def print_sorted_distribution(title, dist):**

    """

    Pretty-print a probability distribution from highest to lowest.

    """

    print(title)

    for name, value in sorted(dist.items(), key=lambda x: x[1], reverse=True):

        print(f"  {name:12s} {value:.4f}")

    print()

**# ============================================================================**

**# MAIN DEMONSTRATION**

**# ============================================================================**

\# This is where the entire scenario is run from beginning to end.

**def main():**

    """

    Run the toy Osprey / tiltrotor inference demonstration.

    """

    # ----------------------------------------------------------------------

    # 1. CHOOSE VECTOR DIMENSION

    # ----------------------------------------------------------------------

    # Higher dimensions make random VSA symbols more cleanly distinct.

    # 512 is a good compromise between clarity and speed for a toy example.

    DIM = 512

    # Create all atomic vectors.

    symbols = make_symbol_table(DIM)

    # ----------------------------------------------------------------------

    # 2. BUILD KNOWN PROTOTYPES

    # ----------------------------------------------------------------------

    prototypes = build_aircraft_prototypes(symbols)

    # ----------------------------------------------------------------------

    # 3. BUILD THE OBSERVATION

    # ----------------------------------------------------------------------

    observation = build_observation(symbols)

    # ----------------------------------------------------------------------

    # 4. DEFINE PRIORS OVER HYPOTHESES

    # ----------------------------------------------------------------------

    # These priors are the agent's initial belief distribution before looking closely at the observation. Interpretable as: "Before seeing the details, how plausible is each known aircraft type?"

    priors = {

        "airplane": 0.30,

        "helicopter": 0.30,

        "blimp": 0.20,

        "ornithopter": 0.20,

    }

    # ----------------------------------------------------------------------

    # 5. SHOW THE KNOWLEDGE AND OBSERVATION

    # ----------------------------------------------------------------------

    print("=" * 78)

    print("TOY PREDICTIVE PROCESSING + VSA DEMO")

    print("=" * 78)

    print()

    print("SCENARIO:")

    print("  The agent sees an unfamiliar aircraft that rises vertically.")

    print("  It does not know the category 'tiltrotor' or 'Osprey'.")

    print("  It must explain the observation using known aircraft priors.")

    print()

    print_feature_list("OBSERVED FEATURES (weighted by observation precision):",

                       observation["features"])

    print("KNOWN PROTOTYPES:")

    print()

    for name, info in prototypes.items():

        print_feature_list(f"Prototype: {name}", info["features"])

    print_sorted_distribution("PRIORS OVER HYPOTHESES:", priors)

    # ----------------------------------------------------------------------

    # 6. COMPARE EACH PROTOTYPE TO THE OBSERVATION

    # ----------------------------------------------------------------------

    # Each prototype acts like a predicted observation.

    # We compare that prediction to the actual observation.

    #

    # This yields:

    #   - similarity

    #   - prediction error

    similarities = {}

    errors = {}

    for name, info in prototypes.items():

        sim, err = compute_prediction_error(

            info["vector"], observation["vector"])

        similarities[name] = sim

        errors[name] = err

    print("PREDICTION MATCH RESULTS:")

    print("  (Higher similarity is better; lower error is better.)")

    print()

    for name in sorted(prototypes.keys()):

        print(

            f"  {name:12s} "

            f"similarity={similarities[name]: .4f}   "

            f"error={errors[name]: .4f}"

        )

    print()

    # ----------------------------------------------------------------------

    # 7. BELIEF UPDATE

    # ----------------------------------------------------------------------

    # We update beliefs by penalizing hypotheses with larger prediction error.

    #

    # beta controls how strongly error influences the posterior.

    # Larger beta -> stronger preference for low-error hypotheses.

    beta = 4.0

    posteriors = posterior_update(priors, errors, beta=beta)

    print(

        f"POSTERIOR BELIEFS AFTER PREDICTION-ERROR UPDATE (beta={beta:.1f}):")

    for name, value in sorted(posteriors.items(), key=lambda x: x[1], reverse=True):

        print(f"  {name:12s} posterior={value:.4f}")

    print()

    # ----------------------------------------------------------------------

    # 8. IDENTIFY THE TOP TWO HYPOTHESES

    # ----------------------------------------------------------------------

    # Test whether a blend of the top two explanations improves fit.

    ranked = sorted(posteriors.items(), key=lambda x: x[1], reverse=True)

    best_name = ranked[0][0]

    second_name = ranked[1][0]

    best_vec = prototypes[best_name]["vector"]

    second_vec = prototypes[second_name]["vector"]

    print("TOP TWO HYPOTHESES TO CONSIDER FOR BLENDING:")

    print(f"  1) {best_name}")

    print(f"  2) {second_name}")

    print()

\# ----------------------------------------------------------------------

    # 9. TEST AN AFFORDANCE-AWARE BLEND (selection only, no synergy)

    # ----------------------------------------------------------------------

    # Use posterior of best hypothesis as alpha so belief informs tie-breaking.

    alpha = posteriors[best_name]

    blend_vec, selected_feats = affordance_blend(

        prototypes[best_name], prototypes[second_name],

        observation, symbols,

        alpha=alpha,

        obs_weight=1.0,

        selective=True, min_role_score=0.0

    )

    # Evaluate the new blend vs observation

    blend_sim, blend_err = compute_prediction_error(

        blend_vec, observation["vector"])

    best_single_sim = similarities[best_name]

    best_single_err = errors[best_name]

    print("BLEND TEST:")

    print(f" blend({best_name}, {second_name}) via role-wise selection")

    print(f" similarity={blend_sim:.4f}")

    print(f" error={blend_err:.4f}")

    print()

    print("SELECTED ROLE–FILLER PAIRS:")

    for r, f, w in selected_feats:

        print(f" - {r:12s} : {f:13s} weight={w:.2f}")

    print()

    print("COMPARISON TO BEST SINGLE HYPOTHESIS:")

    print(f" best single = {best_name}")

    print(f" similarity={best_single_sim:.4f}")

    print(f" error={best_single_err:.4f}")

    print()

    # ----------------------------------------------------------------------

    # 10. INTERPRET THE RESULT

    # ----------------------------------------------------------------------

    if blend_err < best_single_err:

        print("INTERPRETATION:")

        print("  The blended hypothesis explains the observation better than")

        print("  the best single stored prototype.")

        print()

        print("  In PP-style terms:")

        print("    - no single familiar prior fully accounts for the observation")

        print("    - residual prediction error remains under the best pure hypothesis")

        print("    - combining compatible structure from two hypotheses reduces error")

        print()

        print(" In VSA terms:")

        print(" - each hypothesis is a structured vector bundle of role–filler bindings")

        print(" - blending performs affordance selection: for each role, choose the filler")

        print(

            "   from the candidate prototypes that best matches the observation (with a")

        print("   mild posterior bias for tie-breaking)")

        print(" - bundle the selected role–filler bindings into a single hybrid vector")

        print(" - compare the hybrid directly to the observation via cosine similarity")

        print()

        print("  In scenario terms:")

        print("    - the object looks helicopter-like in vertical lift")

        print("    - and airplane-like in body form / implied cruise structure")

        print("    - so a helicopter-airplane hybrid is a better explanation")

    else:

        print("INTERPRETATION:")

        print(" The best stored single prototype already explains the observation")

        print(" as well as or better than the selection-based blend.")

        print(" In this run, selecting affordances from multiple hypotheses did not")

        print(" add explanatory value.")

    print()

    print("=" * 78)

    print("END OF DEMO")

    print("=" * 78)

**# ============================================================================**

**# PYTHON ENTRY POINT**

**# ============================================================================**

\# This is standard Python syntax.

\#

\# When a file is run directly, Python sets __name__ == "__main__".

\# That lets us say:

\#   "If this script is being executed directly, run main()."

\#

\# If the file were imported by another Python file, main() would not run

\# automatically.

if __name__ == "__main__":

    main()

# 4 Supplemental Literature Cited

Friston, Karl. 2010. “The Free-Energy Principle: A Unified Brain Theory?” *Nature Reviews Neuroscience* 11(2):127–38. doi:10.1038/nrn2787.

Furlong, P. Michael, and Chris Eliasmith. 2023. “Bridging Cognitive Architectures and Generative Models with Vector Symbolic Algebras.” *Proceedings of the AAAI Symposium Series* 2(1):262–71. doi:10.1609/aaaiss.v2i1.27686.

Kanerva, Pentti. 2009. “Hyperdimensional Computing: An Introduction to Computing in Distributed Representation with High-Dimensional Random Vectors.” *Cognitive Computation* 1(2):139–59. doi:10.1007/s12559-009-9009-8.

Plate, T. A. 1995. “Holographic Reduced Representations.” *IEEE Transactions on Neural Networks* 6(3):623–41. doi:10.1109/72.377968.
